# Perioperative assessment and management of frailty in elderly patients: a national survey of Italian anesthesiologists

**DOI:** 10.1186/s44158-025-00231-4

**Published:** 2025-02-22

**Authors:** Massimiliano Greco, Ersilia Luca, Fernando Chiumiento, Astrid U. Behr, Gabriella Bettelli, Elena Bignami, Massimo Antonelli, Maurizio Cecconi, Paola Aceto

**Affiliations:** 1 Department of Biomedical Sciences, Humanitas University, Pieve Emanuele, Milano, Italy; 2 Department of Anesthesia and Intensive Care, IRCCS Humanitas Research Hospital, Rozzano, Italy; 3 Dipartimento di Scienze dell’Emergenza, Anestesiologiche e della Rianimazione, Fondazione Policlinico Universitario A. Gemelli IRCCS, Largo A. Gemelli, 8, Rome, 00168 Italy; 4 Università Cattolica del Sacro Cuore, Rome, Italy; 5 ASL Salerno, Salerno, Italy; 6 Department of Anesthesiology and Intensive Care, ULSS6 Euganea, Padua, Italy; 7 Past Director Geriatric Surgery Area and Anaesthesia Dpt, INRCA (Italian National Research Centre on Aging), Ancona, Italy; 8 Unit of Anesthesiology, Division of Critical Care and Pain Medicine, Department of Medicine and Surgery, University of Parma, Parma, Italy

**Keywords:** Frailty assessment, Elderly patients, Survey, Frail patient, Anesthesia, Delirium

## Abstract

**Background:**

The prevalence of frailty is increasing as the global population continues to age. Frailty is associated with poor perioperative outcomes including increased morbidity and mortality. The purpose of this study is to examine current practices and perspectives of anesthesiogists in Italy on the provision of care for elderly surgical patients with frailty.

**Methods:**

We conducted a national survey. Data were collected via an online questionnaire distributed by the Italian Society of Anaesthesia, Analgesia, Resuscitation and Intensive Care (SIAARTI). Responses were collected over 24 weeks between October 2022 and March 2023.

**Results:**

Seven-hundred thirteen anesthetists completed the survey. A total of 39.8% (277) of respondents were working in university hospitals. Frailty scoring was routinely performed in 51.8% of care settings. Only 26.3% of organizations surveyed had a dedicated pathway for perioperative management of frail elderly patients. The most common method for frailty assessment was the subjective assessment by the anesthesiologist (58.3%). More than half of the participants reported the use of ERAS items in most cases. Almost half of respondents reported the use of postoperative screening tools for delirium (45% of respondents).

**Discussion:**

While these results point to the resistance to clinical implementation of frailty assessment, they also highlight the perceived need for careful management. This can help in identifying elderly patients who may require targeted perioperative management and in ensuring the preservation of cognitive and functional status.

**Supplementary Information:**

The online version contains supplementary material available at 10.1186/s44158-025-00231-4.

## Introduction

The increasing prevalence of surgical procedures among elderly patients represents a challenge for anesthesiologists and perioperative care teams. Although surgery is generally safe with a mortality rate below 2%, nearly 80% of postoperative morbidity and mortality occurs in a high-risk group, which includes older, multi-morbid, frail individuals [1], prompting further research to better identify these high-risk patients.

Frailty is a relatively novel adjunct to traditional preoperative assessment that can better stratify the perioperative risk of elderly patients [2, 3]. Despite most experts agree on the need to assess and address frailty before surgery, there are significant disparities in its clinical application, linked to geographic, socioeconomic, center-related, and healthcare professional-related variations [4]. Delirium, loss of autonomy, infection, and cardiac or respiratory problems are extensively reported in frail patients, and frailty is the main determinant of postoperative complications, with a prevalence of 19.2% in elderly surgical patients [5]. Consequently, the early identification of elderly frail patients and appropriate intra- and postoperative management is crucial to reduce perioperative complications. Despite the literature on frailty in surgical patients, there is insufficient understanding regarding the clinical application of guidelines and recommendation, as well as the characteristics of perioperative protocols dedicated to frail patients in Italian hospitals.

Therefore, this study aimed to evaluate anesthesiologists’ current practice and perceptions on perioperative care for elderly frail patients undergoing surgery, from preoperative risk stratification to hospital discharge. In particular, we conducted a nationwide survey to (1) explore the methods used in perioperative medicine to identify frailty, (2) examine targeted interventions in clinical practice, and (3) establish if significant differences in perspectives exist between different types of hospitals.

## Methods

### Setting, participants, and recruitment

We conducted a national survey, after Italian Society of Anaesthesia Analgesia Reanimation and Intensive Care (SIAARTI) research committee approval. The survey was delivered by email to all members of SIAARTI national society, using a link (open survey) within a presentation email, explaining the nature and purpose of the study. IRB approval was not required for this study. Written informed consent for each participating physician was not deemed necessary, as researchers received anonymized data through the national society research office and did not collect or have access to personal data. Reminders were sent by SIAARTI via email to all registered members at weekly intervals from the start to the conclusion of the annual member feedback survey. Duplicate responses were tracked by email address and removed by the SIAARTI research office before being shared with the authors. The survey was conducted over 6 months from October 2022 to March 2023. Study data were collected using an online tool (SurveyMonkey (2024), *Survey tool*, Momentive Inc.)

### Questionnaire design

Participants were queried on the following domains: (1) preoperative assessment (understanding of factors pertinent in the definition of frailty, perceived prevalence, and current assessment of frailty); (2) intraoperative management in frail elderly patients (multimodal monitoring, pharmacological approach/anesthetic strategies, transfusion threshold); (3) postoperative management (application of ERAS items, multidimensional assessment including delirium); and (4) final section including five demographic-geographical questions. Each survey consisted of 21 questions administered to Italian anesthesiologists; *of these, 3 were open-ended, 7 were semi-open-ended, and 10 were closed.*Questions addressing current clinical practice included multiple-choice questions (MCQs), frequency scales, and structured formats. Free comment fields were available to elicit further information where appropriate. Each survey was reviewed for readability and non-ambiguity by experts in frailty care. Checklist for Reporting Results of Internet E-Surveys (CHERRIES) checklist was employed and is included in supplemental material [6].

The questionnaire was developed by SIAARTI’s anesthesia for frail patients section to evaluate current practices and gaps in anesthesia for elderly frail patients, incorporating insights from literature reviews and discussions with group members. It was pretested by board anesthesiologists to refine clarity and flow. The estimated time to complete the survey was 20 min. Supplemental material 1 reports the survey questionnaire.

### Statistics

Statistical analysis was conducted using R (4.3.1), by means of *unipivotr* and *tidyverse* packages. We reported ordinal and continuous variables as mean and standard deviation (SD) or median and interquartile range (IQR) as appropriate, while numbers (percentages) were used for categorical variables. *χ*^2^ tests were used to compare categorical data between groups. The level of experience of respondents was categorized between over 10 years vs. less than 10 years of experience, and hospitals were divided between university hospitals and other centers. A *p*-value < 0.05 was deemed statistically significant.

## Results

### Demographic-geographical data

A total of 713 individuals from 388 hospitals in 187 cities participated in the survey conducted between October 19, 2022, and March 28, 2023. Respondents had different levels of expertise, with university hospitals employing a greater number of in-training residents and recently specialized colleagues, as reported in Table 1. Considering a total of about 9000 SIAARTI member, the response rate was 8%.

**Table 1:** Expertise and hospital characteristics of respondents

Work experience	Overall	Nonteaching hospital	Teaching hospital
In training	96 (13%)	22 (5.0%)	74 (27%)
< 5 years	156 (22%)	101 (23%)	55 (20%)
5 to 10 years	79 (11%)	53 (12%)	26 (9.4%)
10 to 20 years	197 (28%)	130 (30%)	67 (24%)
20 to 30 years	120 (17%)	86 (20%)	34 (12%)
> 30 years	65 (9.1%)	44 (10%)	21 (7.6%)

**Fig. 1:**
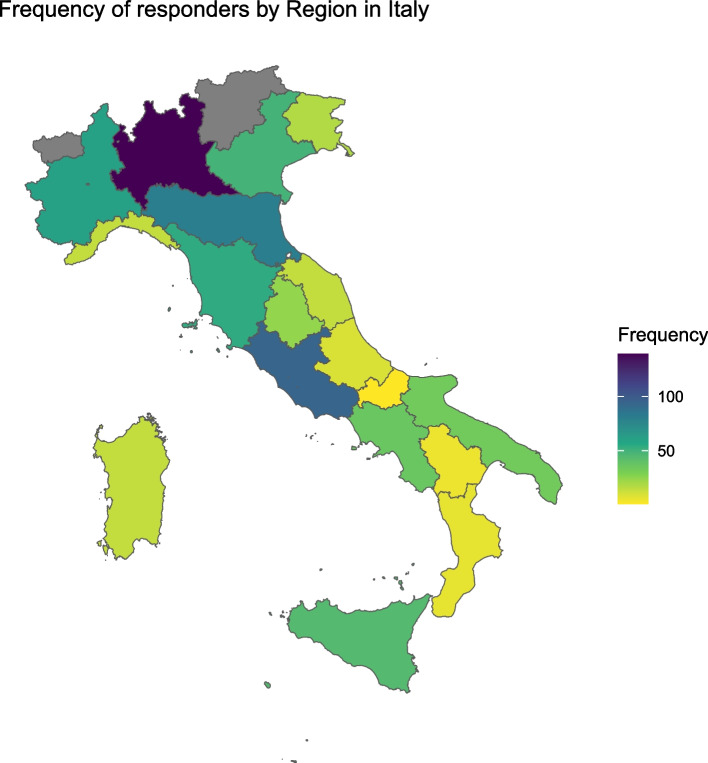
Frequency of respondents by region in Italy

The majority of hospitals were medium-sized hospitals between 200 and 500 beds (35.2%), while 154 hospitals (21.6%) had more than 1000 beds. A total of 39.8% (277) of respondents were working in university hospitals. Figure 1 reports the distribution of respondents in Italian regions, with the majority hailing from Lombardy (139 respondents) and Lazio (94 respondents).

### Preoperative assessment

**Fig. 2:**
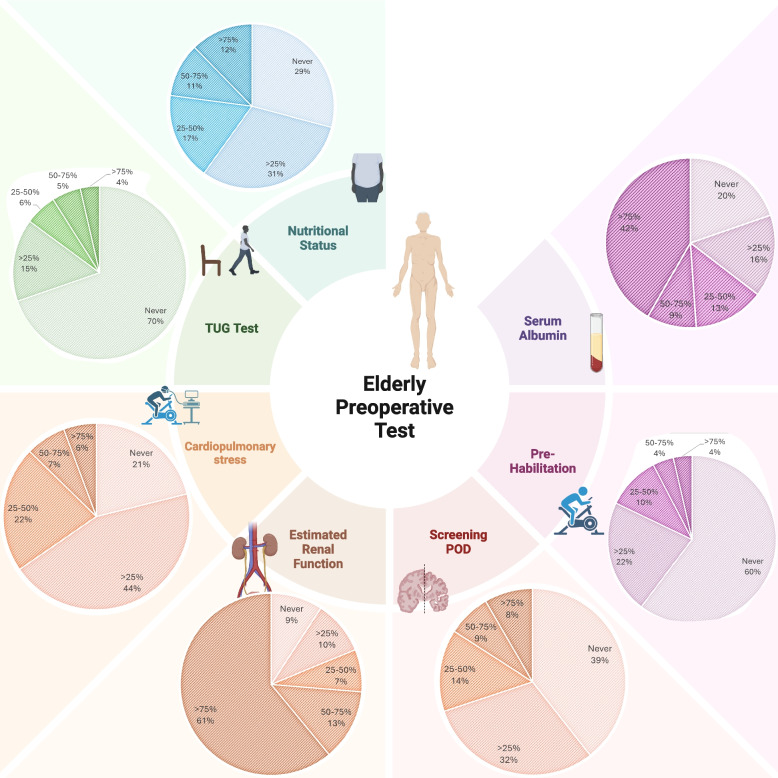
Type and frequency of preoperative testing. Cardiopulmonary stress assessment includes exercise testing (CPET), a diagnostic modality used to evaluate a patient’s functional capacity. The test can provide better understanding of heart and lung function while aiding in the prescription of individualized therapy and prehabilitation programs

Most respondents, comprising 125 individuals (54.8%), defined elderly patients as patients aged 70 years or older. On the contrary, 48 respondents (21.1%) defined elderly patients as patients over the age of 65, without difference between university and nonuniversity hospitals (*p* = 0.402) nor between senior and younger colleagues (*p* = 0.286). Type and characteristics of preoperative assessment tests are reported in Fig. 2.

Most respondents indicated that frailty was assessed in all elderly patients (118 respondents, 51.8%). Eighty respondents (35.1%) indicated that frailty was assessed only in very old patients or patients with cognitive impairment or reduced motility. Table 2 reports preoperative evaluation of frailty. There was no difference according to experience in the anesthesiologist (*p* = 0.312) nor the type of hospital (*p* = 0.439).

**Table 2:** Preoperative evaluation of frailty

	**Overall (*N* = 288)**	**Nonuniversity hospital *vs* university hospital**	***p*-value**	**Experience** ** < 10 y *vs* > 10 y**	***p*-value**
Definition of elderly					
No univocal definition	20 (8.8%)	12 (8.4%) vs 8 (9.4%)		12 (12%) vs 8 (6.5%)	
> 65 years old	48 (21%)	27 (19%) vs 21 (25%)	0.402	25 (24%) vs 23 (19%)	0.286
> 70 years old	125 (55%)	78 (55%) vs 47 (55%)		54 (52%) vs 71 (57%)	
> 80 years old	35 (15%)	26 (18%) vs 9 (11%)		13 (13%) vs 22 (18%)	
In which patients is frailty evaluated in your hospital					
All elderly patients	118 (52%)	75 (52%) vs 43 (51%)		50 (48%) vs 68 (55%)	
Elderly with cognitive impairment	20 (8.8%)	15 (10%) vs 5 (5.9%)	0.439	8 (7.7%) vs 12 (9.7%)	0.312
Elderly unable to walk	22 (9.6%)	15 (10%) vs 7 (8.2%)		14 (13%) vs 8 (6.5%)	
Elderly above 80 years old	38 (17%)	23 (16%) vs 15 (18%)		20 (19%) vs 18 (15%)	
Other	30 (13%)	15 (10%) vs 15 (18%)		12 (12%) vs 18 (15%)	
How do you evaluate frailty					
There is a dedicated protocol for elderly frail patients evaluation	14 (6.1%)	11 (7.7%) vs 3 (3.5%)		3 (2.9%) vs 11 (8.9%)	
With dedicated scales such as CFS and EFS*	53 (23%)	35 (24%) vs (18%)	0.489	26 (25%) vs 27 (22%)	0.031
It is subjectively assessed by anesthesiologist	133 (58%)	84 (59%) vs 49 (58%)		67 (64%) vs 66 (53%)	
With comprehensive geriatric assessment	24 (11%)	13 (9.1%) vs 11 (13%)		6 (5.8%) vs 18 (15%)	
In your hospital, is there a diagnostic-therapeutic plan for perioperative management of frail elderly patients?					
Yes, for frail elderly patients	26 (11%)	14 (9.8%) vs 12 (14%)			
Yes, for elderly patients	9 (3.9%)	5 (3.5%) vs 4 (4.7%)			
Yes, for frail patients	11 (4.8%)	7 (4.9%) vs 4 (4.7%)	0.298		
No, and I don’t think it could help	9 (3.9%)	5 (3.5%) vs 4 (4.7%)			
No, but I believe it could help	168 (74%)	111 (78%) vs 57 (67%)			
Other	5 (2.2%)	1 (0.7%) vs 4 (4.7%)			

^*^CFS clinical frailty scale, *EFS* Edmonton Frail Scale

Supplemental Fig. 2 reports the frequency of elderly frail patients by type of surgery. Elderly frail patients were more prevalent in hip fractures and in vascular and emergency surgery.

**Fig. 3:**
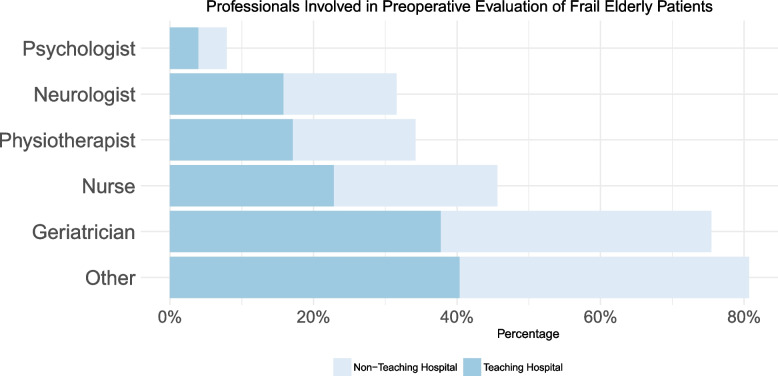
Other specialist involved in frailty evaluation, according to hospital type

In most centers, respondents reported no policy regarding the perioperative care of frail or elderly patients (73.7% respondents), with no differences between university hospitals and nonuniversity hospitals (*p* = 0.298). Beside anesthesiologists and surgeons, the most frequently employed professionals for patient evaluation were geriatricians and nurses (Fig. 3), with a prevalence for nurses or other specialists in nonacademic settings.

The most common method for frailty assessment was the subjective assessment by the anesthesiologist (58.3%), without difference between university and nonuniversity hospitals. Up to 23.2% of respondents reported using clinical frailty scales or Edmonton Frail Scale, while only 10.5% reported the use of CGA. There was a higher prevalence of CGA use (15% vs 5.9%), a higher prevalence of dedicated assessment protocols (8.9% vs 2.9%), and lower subjective assessment (53% vs 64%) for more experienced anesthesiologists compared to younger anesthesiologists (*p* = 0.031). A majority of respondents (55.5%) reported the absence of a dedicated postoperative patient assessment service from anesthesia, while 12.6% demanded re-evaluation to the acute pain service (APS). APS was more common in university hospitals than in nonuniversity hospitals (21% vs 8.1%, *p* = 0.018).

The most common evaluation scales in elderly frail patients undergoing surgery were those concerning estimated renal function (with 61% respondents reporting more than 75% use) and serum albumin (41.7% reporting > 75% use) (Supplemental material 2).

### Intraoperative management

The most employed intraoperative monitoring systems were EEG-based monitoring systems, with 46.4% of respondents reporting its use in at least three out of four cases, and core temperature, which was used by 39.4% of respondents in at least 75% of cases. Neuromuscular transmission monitoring was reported to be used in three out of four cases by 33% of respondents. Neuromuscular blocking agents with specific antagonists, such as rocuronium, were highly used, with 88% of respondents reporting to use rocuronium in most cases and 71% of respondents reporting a high frequency of use of sugammadex (Supplemental material 3, Table S2).

The most common transfusion threshold in elderly frail patients was 8 g/dl, reported by 51% of participants, with no difference between university and nonuniversity hospitals. There was a significant shift toward higher transfusion thresholds when considering senior anesthesiologists compared to younger colleagues, with 22% anesthesiologist with over 10 years of experience preferring a threshold of 9 g/dl compared to 13% in younger colleagues and 10% in experienced vs 2% when considering 10 g/dl as a threshold (*p* = 0.019). (Supplemental material 3, Table S3).

When evaluating anesthetic approaches for open abdominal surgery, the majority of respondents preferred general anesthesia without locoregional anesthesia. This technique was used in three out of four cases by almost half of the respondents, and its usage was even more prevalent in laparoscopic abdominal surgery, where it was used in the majority of cases without fascial or epidural blocks by more than half of the participants. Spinal anesthesia was the preferred technique for hip fracture in elderly frail patients, as it was employed in the large majority of cases by most respondents (Supplemental Fig. S2).

Benzodiazepines were infrequently employed in the premedication of anesthesia. Fentanyl and remifentanil were the main i.v. drugs employed for intraoperative analgesia. Paracetamol was the predominant medicine utilized for patient anesthesia, and the multimodal analgesic approach was selected by 50% of participants as the preferred approach (Supplemental Fig. S3).

### Postoperative management

We additionally assessed the application of ERAS items to elderly frail patients. Active intraoperative warming and pre- and postoperative warming were reported as the most prevalent ERAS intervention used in these patients, with more than half of participants reporting the use of these items in most cases. The next most frequently reported item was postoperative nausea and vomiting (PONV) prophylaxis, which was reported by half of the participants as used in more than three out of four cases.

Almost half of respondents reported using postoperative screening tools for delirium (45% of respondents), even if 19% reported using them only in patients with risk factors. There were no differences according to the type of hospital or the experience of the anesthesiologist. (Supplemental material 4, Table S4). The large majority of hospitals did not have a dedicated postoperative anesthesiological service for re-evaluation of surgical patients (55%), with 17% of respondents reporting postoperative evaluation in case of complications and 13% reporting an involvement through the acute pain service.

## Discussion

We reported the first national survey in Italy on the perioperative management of elderly frail patients, from the preoperative evaluation to the end of hospitalization. Frail patients are becoming increasingly central in modern anesthesia, as there is a growing number of elderly and frail patients undergoing surgery.

Frailty is a concept developed in geriatric medicine, which has gained more and more prominence in perioperative medicine [7]. Frailty is traditionally defined as a syndrome characterized by diminished physiological reserve and heightened vulnerability to stressors. Compared to age and comorbidities, frailty is better associated with perioperative complications and mortality [8]. As a result, guidelines and recommendations have been published incorporating frailty as a criterion to identify patients at elevated risk of unfavorable outcomes, on which value-based healthcare can reduce complications [9]. Timely identification of subpopulations at elevated risk for adverse outcomes, including inhospital mortality loss of autonomy and discharge to long-term care, could enhance patient decision-making and resource allocation in a value-based healthcare framework [10].

Despite the expanding literature and guidelines, there remains a limited understanding of real-world perioperative management and pathways of elderly frail patients undergoing surgery in Italy. Our survey included more than 700 anesthesiologist anesthesiologists in Italy, with approximately equal distribution between university and nonuniversity hospitals, and good stratification in terms of respondents’ age and hospital size.

### Preoperative assessment

Identifying elderly and frail patients is crucial to provide timely risk-reducing interventions. Our results indicate that in Italy, frail patients are predominantly identified by the anesthesiologists (60% of cases), with no significant variation related to hospital characteristics. Age is commonly acknowledged as an insufficient predictor of perioperative complications, a fact reflected in our data. Regardless of the majority of respondents (52%) assessing frailty in elderly patients, only 20% reported using established frailty scales recommended by guidelines, such as the clinical frailty scale or Edmonton Frail Scale [11, 12], with a negligible proportion employing comprehensive geriatric assessment (CGA). Level of expertise nor type of hospital influenced these decisions, while one-third of respondents reported assessing frailty only in very high-risk population such as extremely elderly or obviously frail patients. Although we did not extensively explore the barriers to using validated tools for frailty assessment, several factors may have influenced this issue. A lack of training on the use of specific assessment tools, along with an underappreciation for the importance of employing validated and standardized tools in place of subjective assessment, can have a major role. Despite possible influence of time constraints in preoperative evaluation, the predominance of subjective assessments by respondents might also reflect deficiencies in dedicated tools in electronic or paper health records and poor communication about frailty among healthcare professionals. Increasing training of clinicians but also improving electronic and paper health record could address these issues and should be suggested by best practices in the field. Additionally, our results show large variation in how elderly patients are defined, irrespective of frailty, with the majority of respondents categorizing elderly patients as those aged over 70 years. This deviates from the traditional age criterion of 65 years old to define elderly patients [13] yet aligns with more recent recommendations and national health system guidelines proposing 70 years as the revised threshold. The lack of consensus on establishing a specific age cutoff (65 vs. 70 years) can impact clinical decision-making for elderly frail patients, particularly when defining dedicated perioperative pathways. However, the generally improved health of seniors at 65, owing to better living conditions and healthcare, suggests to reconsider and potentially raise this threshold. This shift parallels trends in retirement age, where an increasing number of 65 years old remain employed across most European countries, and retirement ages are progressively rising. A consensus reflecting these changes is urgently needed. Orthopedic surgeries, especially hip arthroplasties, and urgent or vascular procedures were reported as the surgical areas where elderly and frail patients are more often encountered, with most respondents reporting above 75% or above 50% of patients are frail in these settings. This aligns with previous literature, which traditionally recognizes hip fractures as surgeries performed on elderly frail patients, which are also largely represented in prosthetic orthopedic surgery. Frail patients were frequently observed in emergent-urgent surgery and vascular surgery, a conclusion corroborated by prior research [14].

In addition to anesthesiologists and surgeons, nurses were frequently involved in perioperative evaluation, as were geriatricians when available.

### Intraoperative period

Identification of frail patients is fundamental to reduce perioperative complications, through tailored perioperative monitoring and anesthesiological strategies that might effectively mitigate such issues. Prolonged deep anesthesia and burst suppression have long been linked to postoperative delirium [15], with elderly patients also more at risk for unplanned deep anesthesia [16]. Processed EEG and multiparametric EEG analysis (like density spectra array) have been recommended to reduce delirium [17, 18]. Respondents were aware of these issues, with almost half of the respondents reporting the use of processed EEG and anesthesia depth monitoring in three out of four cases in elderly frail patients.

Monitoring core temperature in older individuals under anesthesia is crucial due to their heightened susceptibility to temperature dysregulation. Advancing age results in physiological alterations such as decreased muscle mass, metabolic and hormonal dysregulation, and compromised vascular responsiveness which affect anesthetic management [19]. Geriatric patients are also more susceptible to detrimental consequences of hypothermia [20], and the implementation of core monitoring among patients was widely acknowledged in our cohort.

There is a large debate regarding the optimal anesthesiological strategies or combinations of strategies in elderly frail patients. Multimodal and blended anesthesia strategies have become increasingly diffuse, with the proliferation of Enhanced Recovery After Surgery (ERAS) strategies and more recently with the availability of ultrasound-guided locoregional anesthesia [21]. Integration of different anesthesiologic approaches may provide several advantages. Epidural anesthesia may mitigate delirium by enhancing analgesia, decreasing opioid usage, and attenuating the stress reaction to surgical procedure. Blended techniques have been proven advantageous in reducing postoperative pain, opioid consumption, and opioid-related delirium [22]. Nonetheless, respondents rarely reported blended or combined anesthesia for abdominal surgery and laparoscopic surgery. The limited utilization of epidural and neuraxial blocks in abdominal surgery may be related not only to complications due to aging and anticoagulant-antithrombotic medications in neuraxial blocks in elderly patients but also to organizational problems and costs. Further analysis is necessary to assess the limitations of neuraxial techniques in this population in Italy. The diffusion of ultrasound-guided trunk blocks has led to a new approach to multimodal anesthesia and analgesia, but these techniques are under-represented among respondents. Future strategies and methodologies should allocate more resources and efforts to enhance the dissemination of truncal blocks, neuraxial anesthesia, and multimodal treatments in elderly frail patients.

Regarding transfusion threshold, even if a restrictive transfusion strategy is generally considered safe for elderly surgical patients [23], frail patients may benefit from a more liberal approach [24]. However, the optimal transfusion strategy remains debated, as there is wide variation in literature on both the definition of the ideal threshold in this population, as well as the criteria distinguishing a restrictive transfusion approach from a liberal one [25]. Respondents identified 8 g/dl as the predominant transfusion cutoff in this population, with experienced anesthesiologists preferring more liberal transfusion strategies compared to younger colleagues.

### Postoperative management

Postoperative warming, effective pain management, and screening for delirium are pivotal components of postoperative medicine in geriatric anesthesia. Most guidelines recommend implementing postoperative screening tools for delirium [9]. Multiple screening techniques exist, each varying in sensitivity, specificity, and predictive values, with the 4-AT test being one of the most frequently utilized [26]. In this study, less than half of the respondents actively utilized screening tools for delirium. This may stem from a lack of awareness about the significant impact delirium can have on patient outcomes. Increasing training for doctors and healthcare professionals during the perioperative period could address this issue. Additionally, the adoption of paper or electronic health records that require delirium detection, alongside with other nudge strategies, could prove beneficial. Establishing national good clinical practices and internal protocols may also help reduce variability in the recognition and management of delirium across different institutions. Risk factors for delirium should be assessed during the preoperative period to identify individuals at increased risk, including those exhibiting depressive symptoms or with visual or auditory impairments [27]. Considering pain management, the relationship between opioid use, pain, and delirium is complex and multifaceted, characterized by a U-shaped correlation where both inadequate pain management and excessive opioid use can precipitate delirium [28]. In this context, multimodal analgesia and opioid-sparing techniques are even more valuable to reduce postoperative delirium and improve postoperative mobility.

### Need for educational activities on evaluation and management of frail elderly patients

This survey highlights the necessity for improved understanding and education opportunities for anesthesiologists in Italy regarding the perioperative care of frail elderly patients. The Italian Society of Anesthesia, Analgesia, Resuscitation, and Intensive Care (SIAARTI) has recently created a section dedicated to perioperative management of frail patients to advocate for best practices in the sector. The frail section, under the auspices of SIAARTI, is offering both in-person and online educational programs and is actively promoting measures to formulate and standardize national guidelines to enhance the quality of care. These initiatives aim to improve the clinical treatment and outcomes of frail elderly patients by providing anesthesiologists with the necessary resources and knowledge for delivering evidence-based and updated care, as well as ways to introduce new therapeutic pathways on this relevant category of patients within each hospital center.

### Limitations

This survey has several limitations. First, when compared to the whole population of anesthesiologists in Italy, the response rate was relatively low, limiting the generalizability of the results. Nonetheless, we reached a good geographical distribution as well as a good distribution of hospital type and age categories of respondents. We cannot exclude biases between respondents and nonrespondents to this survey, in terms of age, type of hospital, expertise, and clinical practice. This is a common limitation in survey conducted via email among a large number of physicians. The total number of participants, exceeding 700, is analogous to comparable surveys. Our survey included mainly SIAARTI members as the main national scientific society in anesthesia and intensive care representing a large number of anesthesiologists in Italy. Nonetheless, members of SIAARTI may differ from nonmembers in terms of experience, knowledge, or practice, aspects that could influence our findings.

Missing responses were relatively common, particularly in questions that appeared further into the questionnaire, which is a typical pattern as respondents tend to lose engagement over time.

## Conclusions

We presented the first national survey in Italy considering the perioperative management of elderly frail patients, revealing that anesthesiologists recognize frailty as a common and significant concern. Our findings emphasize an urgent need to improve frailty assessment using standardized and validated tools and highlight the ambiguity in distinguishing frailty from age-related illnesses among healthcare practitioners. The role of anesthesiologist is central in this setting. While several strategies demonstrated large diffusion, including anesthesia-depth monitoring and temperature management, preoperative and postoperative strategies still present significant opportunities for improvement, as well as multimodal strategies for anesthesia and pain management. There is a need for educational initiatives and clinical policies to improve perioperative care for elderly frail patients, a need to which the national anesthesia society SIAARTI is actively responding through dedicated initiatives.

## Supplementary Information

Additional file 1. Supplemental 1: Study questionnaire. Supplemental material 2: PREOPERATIVE SECTION. Table S1: Preoperatively Tests/exams for major abdominal surgery in elderly frail patients. Figure S1: Percentage of elderly frail patients by surgical area. Supplemental material 3: INTRAOPERATIVE MANAGEMENT. Table S2: intraoperative monitoring techniques. Table S3: transfusion thresholds. Table S4: Use of myorelaxant and antagonists. Figure S2: anesthesia therapeutic strategies in open abdominal surgery, laparoscopic surgery, and hip fracture. Figure S3: intraoperative anesthesia and analgesia, postoperative analgesia strategies in elderly frail patients. Figure S4: Use of Enhanced Recovery After Surgery items in elderly frail patients. Supplemental material 4: POSTOPERATIVE MANAGEMENT: Table S5: Delirium assessment scales, other perioperative assessment scales, and postoperative service in elderly frail patients. Supplemental 5: Checklist for Reporting Results of Internet E-Surveys (CHERRIES)

## Data Availability

Data is available from the corresponding author upon request.
