# Supplementary material for: Perioperative assessment and management of frailty in elderly patients: a national survey of Italian anesthesiologists
Source: J Anesth Analg Crit Care. 2025 Feb 22;5:11. doi: 10.1186/s44158-025-00231-4 (PMC11846183; doi:10.1186/s44158-025-00231-4)
Supplement: Supplementary file 1 — Additional file 1. Supplemental 1: Study questionnaire. Supplemental material 2: PREOPERATIVE SECTION. Table S1: Preoperatively Tests/exams for major abdominal surgery in elderly frail patients. Figure S1: Percentage of elderly frail patients by surgical area. Supplemental material 3: INTRAOPERATIVE MANAGEMENT. Table S2: intraoperative monitoring techniques. Table S3: transfusion thresholds. Table S4: Use of myorelaxant and antagonists. Figure S2: anesthesia therapeutic strategies in open abdominal surgery, laparoscopic surgery, and hip fracture. Figure S3: intraoperative anesthesia and analgesia, postoperative analgesia strategies in elderly frail patients. Figure S4: Use of Enhanced Recovery After Surgery items in elderly frail patients. Supplemental material 4: POSTOPERATIVE MANAGEMENT: Table S5: Delirium assessment scales, other perioperative assessment scales, and postoperative service in elderly frail patients. Supplemental 5: Checklist for Reporting Results of Internet E-Surveys (CHERRIES) [file 44158_2025_231_MOESM1_ESM.docx]

**Supplemental 1: Study questionnaire**

**Questionnaire**

**SECTION A:** **PREOPERATIVE ASSESSMENT**

**1. How do you define elderly patient in your center?** (only one correct answer)

- Patient over 65 years old
- Patient over 70 years old
- Patient over 80 years old
- It is not univocally defined

**2. In which elderly patients do you evaluate frailty in your hospital?**

- In all elderly patients
- In elderly patients unable to ambulate independently
- In elderly patients with cognitive impairments
- In elderly patients over 80 years old
- Other

**3. How do you assess frailty?**

- Using a specific protocol for frail patients
- Using specific screening tests administered in the preoperative assessment (Clinical Frailty Scale, Edmonton Frail Scale)
- Using Comprehensive Geriatric Assessment (CGA)
- It is subjectively reported by the anaesthesiologist
- Other

**4. At your hospital, what is the percentage of elderly frail patients undergoing surgery in one of the following areas?** *(Matrix of answers)*

|  | <25% | 25-50% | 50-75% | >75% |
| --- | --- | --- | --- | --- |
| **Abdominal surgery** |  |  |  |  |
| **Thoracic surgery** |  |  |  |  |
| **Cardiac surgery** |  |  |  |  |
| **Vascular surgery** |  |  |  |  |
| **Day surgery** |  |  |  |  |
| **Emergency surgery** |  |  |  |  |
| **Hip fracture** |  |  |  |  |
| **Other orthopedic surgery** |  |  |  |  |

**5. In your hospital, is there a defined diagnostic-therapeutic pathway or hospital policy regarding the perioperative care of elderly frail patients?** (only one correct answer)

- - There is a plan for elderly patients
  - There is a plan for frail patients
  - There is a plan for elderly frail patients
  - There is no plan, but I believe it would be useful to develop one in my hospital
  - There is no plan, but I do not think it could be useful
  - Other

**6. Which of the following professionals are involved in the preoperative assessment of elderly frail patients at your hospital, together with the anesthetist and surgeon**? (more than one correct answer)

- - Geriatrician
  - Neurologist
  - Physiotherapist
  - Nurse
  - Psychologist
  - Other

**7. In what percentage of elderly frail patients scheduled for major abdominal surgery do you assess the following tests/exams preoperatively?** *(matrix of answers)*

|  | **Never** | **<25%** | **25-50%** | **50-75%** | **>75%** |
| --- | --- | --- | --- | --- | --- |
| **Albumin (serum levels)** |  |  |  |  |  |
| **Estimate of renal function calculated with eGFR formulas (e.g.: Cockcroft-Gault or MDRD formulas)** |  |  |  |  |  |
| **Cardiopulmonary exercise testing** |  |  |  |  |  |
| **Nutritional status assessment** |  |  |  |  |  |
| **Screening for risk factors for delirium** |  |  |  |  |  |
| **Timed Up and Go test** |  |  |  |  |  |
| **Prehabilitation pathway** |  |  |  |  |  |

**SECTION B****: INTRAOPERATIVE MANAGEMENT**

**8. In what percentage of elderly frail patients undergoing general anesthesia for major abdominal surgery do you use one of the following monitoring techniques to optimize anesthetic management?** *(matrix of answers)*

|  | **Never** | **<25%** | **25-50%** | **50-75%** | **>75%** |
| --- | --- | --- | --- | --- | --- |
| **EEG-based depth of anesthesia monitors** |  |  |  |  |  |
| **Core temperature (bladder or oesophageal)** |  |  |  |  |  |
| **Advanced hemodynamic monitoring (calibrated or uncalibrated pulse contour analysis)** |  |  |  |  |  |
| **Invasive arterial blood pressure monitoring** |  |  |  |  |  |
| **Other** |  |  |  |  |  |

**9. In elderly frail patients undergoing general anesthesia, what type of muscle relaxant and antagonist do you use? Do you use neuromuscular transmission monitoring? Please indicate the percentage of usage.** *(matrix of answers)*

|  | **<25%** | **25-50%** | **50-75%** | **>75%** |
| --- | --- | --- | --- | --- |
| **Myorelaxants with a specific antagonist (Rocuronium or Vecuronium)** |  |  |  |  |
| **Other myorelaxants** |  |  |  |  |
| **Antagonists: Sugammadex** |  |  |  |  |
| **Antagonists: Neostigmine + atropine** |  |  |  |  |
| **Use of neuromuscular transmission monitoring** |  |  |  |  |

**10. What haemoglobin threshold do you use for transfusion of packed red blood cells in elderly frail patients (with no history of ischemic heart disease)?**

- - Transfusion at the threshold of 7 g/dL Hemoglobin
  - Transfusion at the threshold of 8 g/dL Hemoglobin
  - Transfusion at the threshold of 9 g/dL Hemoglobin
  - Transfusion at the threshold of 10 g/dL Hemoglobin
  - Other

**11. In what percentage of elderly frail patients do you use one of the following strategies?** *(more than one correct answer)*

|  | **Never** | **<25%** | **25-50%** | **50-75%** | **>75%** |
| --- | --- | --- | --- | --- | --- |
| **In open abdominal surgery:** |  |  |  |  |  |
| General + epidural anesthesia |  |  |  |  |  |
| General anesthesia + analgesic spinal anesthesia |  |  |  |  |  |
| General anesthesia + abdominal wall block |  |  |  |  |  |
| Regional anesthesia |  |  |  |  |  |
| General anesthesia |  |  |  |  |  |
| **In laparoscopic abdominal surgery:** |  |  |  |  |  |
| General + epidural anesthesia |  |  |  |  |  |
| General anesthesia + analgesic spinal anesthesia |  |  |  |  |  |
| General anesthesia + abdominal wall block |  |  |  |  |  |
| General anesthesia |  |  |  |  |  |
| **Hip fracture:** |  |  |  |  |  |
| Spinal anesthesia |  |  |  |  |  |
| Peripheral nerve blocks |  |  |  |  |  |
| Spinal anesthesia + peripheral block |  |  |  |  |  |
| General anesthesia |  |  |  |  |  |
| General anesthesia + peripheral block |  |  |  |  |  |

**12. Regarding the pharmacological management, what intra- and postoperative strategies do you use in elderly frail patients?**

|  | **Never** | **<25%** | **25-50%** | **50-75%** | **>75%** |
| --- | --- | --- | --- | --- | --- |
| Premedication with benzodiazepines |  |  |  |  |  |
| **In case of intraoperative opioids use, how frequently do you administer the following medications?** |  |  |  |  |  |
| Fentanyl |  |  |  |  |  |
| Remifentanil |  |  |  |  |  |
| Sufentanil |  |  |  |  |  |
| Morphine |  |  |  |  |  |
| How frequently do you use "opioid-free" anesthesia? |  |  |  |  |  |
| **Postoperative analgesia: how often do you use the following strategies?** |  |  |  |  |  |
| Paracetamol |  |  |  |  |  |
| NSAIDs* |  |  |  |  |  |
| Morphine PCA* |  |  |  |  |  |
| Subcutaneous morphine/ iv morphine by continuous infusion |  |  |  |  |  |
| Combination oxycodone/naloxone and opioids per os |  |  |  |  |  |
| Multimodal strategies |  |  |  |  |  |

***** NSAIDS: Non-Steroidal Anti-Inflammatory Drugs, PCA=Patient-Controlled Analgesia

**SECTION C:** **POSTOPERATIVE MANAGEMENT**

**13. In what percentage of elderly frail patients do you apply the following ERAS items?**

|  | **Never** | **<25%** | **25-50%** | **50-75%** | **>75%** |
| --- | --- | --- | --- | --- | --- |
| Routine PONV prophylaxis |  |  |  |  |  |
| Removal of urinary catheter within 24 hours |  |  |  |  |  |
| Active intraoperative warming |  |  |  |  |  |
| Active pre- or postoperative warming |  |  |  |  |  |
| Early postoperative nutrition |  |  |  |  |  |

**14. Do you use postoperative delirium assessment scales?**

- - Yes, from the recovery room and for the first 5 postoperative days
  - Yes, from admission to the ward and for the first 5 postoperative days
  - Yes, but I use them only in patients with risk factors
  - No, I do not use them
  - Other

**15. In your hospital, is there a service or anesthesiologist assigned to the postoperative assessment?**

- - Yes, for all the patients
  - Yes, only for patients with complications
  - Yes, handled by the acute pain service/pain management team
  - No

**16. In elderly patients undergoing surgery, which of the following assessments scales do you use?** *(*

- - Independence level (BADL/IADL scales)
  - Cognitive status (MMSE, Clock Test, Mini-Cog, or other)
  - Nutritional status (MNA, height, weight, BMI, or other)
  - The presence of depression (GDS or other)
  - Visual and hearing impairments and the adequacy of the prosthetic devices in use
  - Assess the availability of familiar and social support
  - None
  - Other

**D) FINAL SECTION**

**17. In which year did you complete residency training in anesthesia?_____________**

**18. In which region/city do you work? _________________________________________**

**19. Name of the hospital where you work:_____________________________________**

**20. Indicate the type of hospital in which you are working**

- - University hospital
  - Non-University hospital
  - Hub Hospital
  - Spoke Hospital
  - Other (please specify)

**21. What is the hospital size in beds ?**

- < 200
- 200-500
- 500-1000
- >1000

**Supplemental material 2: PREOPERATIVE SECTION**

**Table S1: Preoperatively Tests/exams for major abdominal surgery in elderly frail patients**

|  | **Never** | **25%** | **25-50%** | **50-75%** | **>75%** |
| --- | --- | --- | --- | --- | --- |
| **Preoperatively testing for major abdominal surgery in elderly frail patients** | | | | | |
| Serum Albumin levels | 19.7 | 15.7 | 13.5 | 9.4 | 41.7 |
| Prehabilitation | 60.3 | 21.9 | 10.3 | 4 | 3.6 |
| Screening for risk factor for delirium | 39.4 | 30.5 | 14.2 | 7.5 | 8.4 |
| Estimated renal function (eGFR) | 9.2 | 9.6 | 7.5 | 12.7 | 61 |
| Cardiopulmonary exercise testing | 21.1 | 44.3 | 21.9 | 7 | 5.7 |
| Timed Up and Go test | 69.7 | 15.4 | 5.9 | 5.4 | 3.6 |
| Nutritional status assessment | 29.1 | 30.8 | 17.2 | 10.6 | 12.3 |

**Figure S1: Percentage of elderly frail patients by surgical area**

**Supplemental material 3**: **INTRAOPERATIVE MANAGEMENT**

**Table S2: intraoperative monitoring techniques**

|  | **Never** | **25%** | **25-50%** | **50-75%** | **>75%** | **Total** |
| --- | --- | --- | --- | --- | --- | --- |
| **Monitoring techniques to optimize anesthetic management in general anesthesia for major abdominal surgery** | | | | | |  |
| Invasive Arterial Blood Pressure monitoring | 2.1 | 16 | 17 | 30.9 | 34 | 194 |
| Advanced Hemodynamic monitoring (PAC*) | 26.8 | 23.7 | 24.2 | 14.4 | 10.8 | 194 |
| Depth of Anesthesia with EEG | 16 | 14.4 | 12.4 | 10.8 | 46.4 | 194 |
| Core Temperature (bladder/oesophageal) | 15.5 | 17.6 | 11.4 | 16.1 | 39.4 | 193 |

*PAC=pulse contour analysis

**Table S3: transfusion thresholds**

|  | **Overall (N=288)** | **Non-University hospital *vs* University Hospital** | **P value** | **Experience**  **<10y *vs* >10y** | **P value** |
| --- | --- | --- | --- | --- | --- |
| **Transfusion thresholds in patients with no history of ischemic heart disease** | | |  | |  |
| 10g/dl | 12 (6.2%) | 7 (5.5%) vs 5 (7.5%) | 0.606 | 2 (2.2%) vs 10 (9.7%) | 0.019 |
| 9 g/dl | 35 (18%) | 21 (17%) vs14 (21%) |  | 12 (13%) vs 23 (22%) |  |
| 8 g/dl | 98 (51%) | 69 (54%) vs 29 (43%) |  | 48 (53%) vs 50 (49%) |  |
| 7 g/dl | 39 (20%) | 23 (18%) vs 16 (24%) |  | 25 (27%) vs 14 (14%) |  |
| Other | 10 (5.2%) | 7 (5.5%) vs3 (4.5%) |  | 4 (4.4%) vs 6 (5.(%) |  |

**Table S4: Use of myorelaxant and antagonists**

|  | **Never** | **25%** | **25-50%** | **50-75%** | **>75%** | **Total** |
| --- | --- | --- | --- | --- | --- | --- |
| **Type of muscle relaxant and antagonist do you use? Do you use neuromuscular transmission monitoring?** | | | | | | |
| Antagonists: Sugammadex | 0.5 | 11.1 | 4.8 | 12.2 | 71.4 | 189 |
| Antagonists: Neostigmine + atropine | 47.8 | 31.5 | 10.1 | 6.7 | 3.9 | 178 |
| NMBAs with specific antagonist | 1 | 3.1 | 2.1 | 5.7 | 88.1 | 194 |
| Use of neuromuscular transmission monitoring | 16.5 | 17.5 | 16 | 17 | 33 | 194 |
| Other myorelaxants | 55.9 | 32.4 | 4.7 | 2.9 | 4.1 | 170 |

**Figure S2: anesthesia therapeutic strategies in open abdominal surgery, laparoscopic surgery, and hip fracture.**

**Figure S3: intraoperative anesthesia and analgesia, postoperative analgesia strategies in elderly frail patients**

**Figure S4: Use of Enhanced Recovery After Surgery items in elderly frail patients**

**Supplemental material 4**: **POSTOPERATIVE MANAGEMENT**

**Table S5: Delirium assessment scales, other perioperative assessment scales, and postoperative service in elderly frail patients**

|  | **Overall (N=288)** | **Non-University hospitals *vs* University Hospitals** | **P value** | **Experience**  **<10y *vs* >10y** | **P value** |
| --- | --- | --- | --- | --- | --- |
| **Postoperative delirium assessment scales** | | | | |  |
| Yes, from the recovery room and for the first 5 postoperative days | 23 (12%) | 12 (9.7%) vs 11 (16%) |  | 12 (13%) vs 11 (11%) |  |
| Yes, from admission to post op day | 18 (9.4%) | 13 (10%) vs 5 (7.5%) | 0.599 | 10 (11%) vs 8 (7.9%) | 0.673 |
| Yes, only in patients with risk factors | 37 (19%) | 26 (21%) vs 11 (16%) |  | 18 (20%) vs19 (19%) |  |
| Not used | 105 (55%) | 67 (54%) vs 38 (57%) |  | 48 (53%) vs 57 (56%) |  |
| Other | 8 (4.2%) | 6 (4.8%) vs 2 (3.0%) |  | 2 (2.2%) vs 6 (5.9%) |  |
| **Other perioperative assessment scales for postoperative management** | | |  | |  |
| Independence level (BADL/IADL scales) | 58 (15%) | 37 (14%) vs 21 (16%) |  | 22 (14%) vs 36 (15%) |  |
| Cognitive status (MMSE, Clock Test, Mini-Cog, or other) | 58 (15%) | 40 (15%) vs 18 (13%) |  | 24 (15%)vs 34 (14%) |  |
| Nutritional status (MNA, height, weight, BMI, or other) | 84 (21%) | 54 (21%) vs 30 (22%) | 0.977 | 32 (20%) vs 52 (22%) | 0.218 |
| Presence of depression (GDT or other) | 22 (5.6%) | 16 (6.2%) vs 6 (4.4%) |  | 5 (3.1%) vs 17 (7.2%) |  |
| Visual and auditory impairments and adequacy of prostheses in use | 34 (8.6%) | 22 (8.5%) vs 12 (8.9%) |  | 13 (8.2%) vs 21 (8.9%) |  |
| Availability of family and social support | 73 (19%) | 49 (19%) vs 24 (18%) |  | 28 (18%) vs 45 (19%) |  |
| None | 65 (16%) | 41 (16%) vs 24 (18%) |  | 35 (22%) vs 30 (13%) |  |
| **Presence of a service or anesthesiologist for follow-up assessment of patients after surgery** | | | | | |
| Yes, managed by the acute pain service | 24 (13%) | 10 (8.1%) vs 14 (21%) |  |  |  |
| Yes, only in patients with complications | 32 (17%) | 19 (15%) vs 13 (19%) |  |  |  |
| Yes, for all patients | 19 (9.9%) | 16 (13%) vs 3 (4.5%) | 0.018 |  |  |
| Other | 10 (5.2%) | 9 (7.3%) vs 1 (1.5%) |  |  |  |
| No | 106 (55%) | 70 (56%) vs 36 (54%) |  |  |  |

**Supplemental 5**

**Checklist for Reporting Results of Internet E-Surveys (CHERRIES)**

| ***Checklist Item*** | ***Explanation*** | ***Page Number*** |
| --- | --- | --- |
| Describe survey design | Describe target population, sample frame. Is the sample a convenience sample? (In “open” surveys this is most likely.) | 4 |
| IRB approval | Mention whether the study has been approved by an IRB. | 4 |
| Informed consent | Describe the informed consent process. Where were the participants told the length of time of the survey, which data were stored and where and for how long, who the investigator was, and the purpose of the study? | 4 |
| Data protection | If any personal information was collected or stored, describe what mechanisms were used to protect unauthorized access. | 4 |
| Development and testing | State how the survey was developed, including whether the usability and technical functionality of the electronic questionnaire had been tested before fielding the questionnaire. | 5 |
| Open survey versus closed survey | An “open survey” is a survey open for each visitor of a site, while a closed survey is only open to a sample which the investigator knows (password-protected survey). | 4 |
| Contact mode | Indicate whether or not the initial contact with the potential participants was made on the Internet. (Investigators may also send out questionnaires by mail and allow for Web-based data entry.) | 4 |
| Advertising the survey | How/where was the survey announced or advertised? Some examples are offline media (newspapers), or online (mailing lists – If yes, which ones?) or banner ads (Where were these banner ads posted and what did they look like?). It is important to know the wording of the announcement as it will heavily influence who chooses to participate. Ideally the survey announcement should be published as an appendix. | 4 |
| Web/E-mail | State the type of e-survey (eg, one posted on a Web site, or one sent out through e-mail). If it is an e-mail survey, were the responses entered manually into a database, or was there an automatic method for capturing responses? | 4 |
| Context | Describe the Web site (for mailing list/newsgroup) in which the survey was posted. What is the Web site about, who is visiting it, what are visitors normally looking for? Discuss to what degree the content of the Web site could pre-select the sample or influence the results. For example, a survey about vaccination on a anti-immunization Web site will have different results from a Web survey conducted on a government Web site | 4 |
| Mandatory/voluntary | Was it a mandatory survey to be filled in by every visitor who wanted to enter the Web site, or was it a voluntary survey? | 4 |
| Incentives | Were any incentives offered (eg, monetary, prizes, or non-monetary incentives such as an offer to provide the survey results)? | No incentives |
| Time/Date | In what timeframe were the data collected? | 4 |
| Randomization of items or questionnaires | To prevent biases items can be randomized or alternated. | Nd |
| Adaptive questioning | Use adaptive questioning (certain items, or only conditionally displayed based on responses to other items) to reduce number and complexity of the questions. | 4 |
| Number of Items | What was the number of questionnaire items per page? The number of items is an important factor for the completion rate. | nd |
| Number of screens (pages) | Over how many pages was the questionnaire distributed? The number of items is an important factor for the completion rate. | 4 sections |
| Completeness check | It is technically possible to do consistency or completeness checks before the questionnaire is submitted. Was this done, and if “yes”, how (usually JAVAScript)? An alternative is to check for completeness after the questionnaire has been submitted (and highlight mandatory items). If this has been done, it should be reported. All items should provide a non-response option such as “not applicable” or “rather not say”, and selection of one response option should be enforced. | No |
| Review step | State whether respondents were able to review and change their answers (eg, through a Back button or a Review step which displays a summary of the responses and asks the respondents if they are correct). | Yes |
| Unique site visitor | If you provide view rates or participation rates, you need to define how you determined a unique visitor. There are different techniques available, based on IP addresses or cookies or both. | 4 |
| View rate (Ratio of unique survey visitors/unique site visitors) | Requires counting unique visitors to the first page of the survey, divided by the number of unique site visitors (not page views!). It is not unusual to have view rates of less than 0.1 % if the survey is voluntary. | nd |
| Participation rate (Ratio of unique visitors who agreed to participate/unique first survey page visitors) | Count the unique number of people who filled in the first survey page (or agreed to participate, for example by checking a checkbox), divided by visitors who visit the first page of the survey (or the informed consents page, if present). This can also be called “recruitment” rate. | Nd |
| Completion rate (Ratio of users who finished the survey/users who agreed to participate) | The number of people submitting the last questionnaire page, divided by the number of people who agreed to participate (or submitted the first survey page). This is only relevant if there is a separate “informed consent” page or if the survey goes over several pages. This is a measure for attrition. Note that “completion” can involve leaving questionnaire items blank. This is not a measure for how completely questionnaires were filled in. (If you need a measure for this, use the word “completeness rate”.) | nd |
| Cookies used | Indicate whether cookies were used to assign a unique user identifier to each client computer. If so, mention the page on which the cookie was set and read, and how long the cookie was valid. Were duplicate entries avoided by preventing users access to the survey twice; or were duplicate database entries having the same user ID eliminated before analysis? In the latter case, which entries were kept for analysis (eg, the first entry or the most recent)? | nd |
| IP check | Indicate whether the IP address of the client computer was used to identify potential duplicate entries from the same user. If so, mention the period of time for which no two entries from the same IP address were allowed (eg, 24 hours). Were duplicate entries avoided by preventing users with the same IP address access to the survey twice; or were duplicate database entries having the same IP address within a given period of time eliminated before analysis? If the latter, which entries were kept for analysis (eg, the first entry or the most recent)? | Nd |
| Log file analysis | Indicate whether other techniques to analyze the log file for identification of multiple entries were used. If so, please describe. | Nd |
| Registration | In “closed” (non-open) surveys, users need to login first and it is easier to prevent duplicate entries from the same user. Describe how this was done. For example, was the survey never displayed a second time once the user had filled it in, or was the username stored together with the survey results and later eliminated? If the latter, which entries were kept for analysis (eg, the first entry or the most recent)? | Nd |
| Handling of incomplete questionnaires | Were only completed questionnaires analyzed? Were questionnaires which terminated early (where, for example, users did not go through all questionnaire pages) also analyzed? | 5 |
| Questionnaires submitted with an atypical timestamp | Some investigators may measure the time people needed to fill in a questionnaire and exclude questionnaires that were submitted too soon. Specify the timeframe that was used as a cut-off point, and describe how this point was determined. | Nd |
| Statistical correction | Indicate whether any methods such as weighting of items or propensity scores have been used to adjust for the non-representative sample; if so, please describe the methods. | nd |

This checklist has been modified from Eysenbach G. Improving the quality of Web surveys: the Checklist for Reporting Results of Internet E-Surveys (CHERRIES). J Med Internet Res. 2004 Sep 29;6(3):e34 [erratum in J Med Internet Res. 2012; 14(1): e8.]. Article available at [https://www.jmir.org/2004/3/e34](https://www.jmir.org/2004/3/e34/)/; erratum available <https://www.jmir.org/2012/1/e8/>. Copyright ©Gunther Eysenbach. Originally published in the [Journal of Medical Internet](http://www.jmir.org) Research, 29.9.2004 and 04.01.2012.
